# Consumption of chocolate in pregnant women and risk of preeclampsia: a systematic review

**DOI:** 10.1186/2046-4053-2-114

**Published:** 2013-12-20

**Authors:** Jaime Andres Mogollon, Catherine Boivin, Kadhel Philippe, Stéphane Turcotte, Simone Lemieux, Claudine Blanchet, Emmanuel Bujold, Sylvie Dodin

**Affiliations:** 1 Saint-François d’Assise Hospital, Centre hospitalier universitaire de Québec (CHUQ), Québec, Canada; 2 Department of Dermatology, CHUQ, Québec, Canada; 3 University Teaching Hospital, Pointe-A-Pitre, Guadeloupe, France; 4 Saint-François d’Assise Hospital, Clinical Research Platform CHUQ, Research Axis of Public Health and Practice-Changing Research, Québec, Canada; 5 Department of Food Sciences and Nutrition, Institute of Nutrition and Functional Foods (INAF), Université Laval, Québec, Canada; 6 Reproductive Biology Research Center, Centre hospitalier de l'Université Laval (CHUL)-CHUQ, Québec, Canada; 7 Department of Obstetrics and Gynecology, Research Center, Saint-François d’Assise Hospital, CHUQ, Université Laval, Québec, Canada

**Keywords:** Preeclampsia, Pregnant women, Chocolate, Theobromine, Systematic review

## Abstract

**Background:**

Previous studies have been limited in reporting the association between chocolate consumption, measured by interviewer-administered questionnaire or serum theobromine, a biomarker for cocoa, and risk of preeclampsia, and have showed somewhat conflicting results.

**Methods/Design:**

A systematic review of observational and experimental studies will be carried out. We will examine PubMed, Embase, and the entire Cochrane Library. Studies of chocolate consumption compared or not with placebo or low flavanol chocolate during pregnancy will be evaluated to investigate the effect of chocolate consumption in pregnant women on the risk of preeclampsia or pregnancy-induced hypertension. Screening for inclusion, data extraction, and quality assessment will be performed independently by two reviewers in consultation with a third reviewer. Validity of the studies will be ascertained by using the Cochrane Collaboration’s tool. Relative risk of preeclampsia will be the primary measure of treatment effect. Heterogeneity will be explored by subgroup analysis according to confounding factors and bias.

**Discussion:**

This systematic review will contribute to establish the current state of knowledge concerning the possible association between chocolate consumption and prevention of preeclampsia. Furthermore, it will justify if additional experimental trials are necessary to better evaluate the benefits of chocolate consumption on the risk of preeclampsia.

**Trial registration:**

This systematic review has been registered in the PROSPERO international prospective register of systematic reviews. The registration number is: CRD42013005338

## Background

Preeclampsia, a syndrome defined by new-onset gestational hypertension and proteinuria, originates in the placenta and is characterized by generalized maternal dysfunction of the endothelium. Endothelium dysfunction leads to clinical symptoms of the mother [1,2]. Preeclampsia is one of the most common medical disorders affecting pregnancy, with potentially severe consequences for mother and child, particularly in developing countries [3]. It is estimated that 3% to 8% of all pregnancies are affected by this complication. Every year, preeclampsia is responsible for approximately 60,000 deaths worldwide [4]. More than half of women with preeclampsia will undergo Caesarean delivery. Preeclampsia increases the risk of perinatal mortality by five-fold, and is the primary cause of low birth weight in infants [4]. Numerous studies have suggested that women who develop preeclampsia have an increased risk of cardiovascular disease later in life [5]. The cardiovascular manifestations of preeclampsia share many characteristics and risk factors of cardiovascular disease, including hypertension, endothelium dysfunction, and oxidative stress [6,7].

Our recent data confirmed that endothelial function is impaired in women who are at risk of preeclampsia, and occurs before the development of the clinical syndrome [8]. There is strong evidence that maternal nitric oxide (NO) deficiency plays a key role in the development of preeclampsia [8]. Despite intensive research, preeclampsia remains an idiopathic disease for which no effective prophylactic measures are available to patients [3]. Therapeutic approaches focusing on up-regulating NO availability may be useful targets in preeclampsia prevention. Flavanols, the most common flavonoids in dark chocolate, are potent antioxidants capable of inducing NO-dependent vasodilatation. Two recent meta-analyses of randomized controlled trials (RTCs) confirmed that flavanol-rich chocolate has a beneficial influence on endothelial function and reduces systolic and diastolic blood pressure (BP) [9,10]. Theobromine, a major constituent of dark chocolate, also possesses vasodilatation and relaxing smooth tissue properties [11].

As endothelial dysfunction is fundamental to the development of preeclampsia, we therefore hypothesize that flavanol-rich chocolate consumption reduces the risk of preeclampsia via improvement of endothelial function in pregnant women. Theobromine could account for, or contribute to, enhancing the effect of flavanols. Indeed, recently, a few observational and experimental studies have been designed to evaluate the associations between chocolate consumption and/or theobromine, a biomarker of cocoa intake with preeclampsia, but the results were conflicting [12-14]. The primary objective of this systematic review is to investigate the effect of chocolate consumption on preeclampsia risk in pregnant women using observational and experimental studies.

## Methods

Methods to be used for this systematic review have been elaborated as described by the Preferred Reporting Items for Systematic Reviews and Meta-Analyses (PRISMA) statement and according to the recommendations of the *Cochrane Handbook for Systematic Reviews of Interventions*[15].

### Criteria for considering studies for this review

#### Eligibility criteria

The study population, the intervention, and the outcome will be used as criteria for eligibility of studies in this systematic review, reflecting the participants, interventions, comparators, outcomes, and study design (PICOS) approach. All publication years, languages, and publication statuses will be considered as report characteristics. No language, publication date, or publication status restrictions will be imposed.

#### Design of studies

In this systematic review, we will consider RCTs, well-designed quasi-experimental (quasi-RCT) studies, controlled before and after (CBA) studies, interrupted time series (ITS) analyses, and observational studies (cohort, case–control, and descriptive).

#### Participants

Pregnant women will be considered in this systematic review. Studies concerning all women without any age restriction and with a live fetus will be eligible for participation in this systematic review.

#### Interventions

Studies evaluating consumption of chocolate by either interviewer-administered questionnaire or serum theobromine will be considered in this systematic review. No treatment period restrictions will be imposed. All studies of chocolate consumption, regardless of type or dose of chocolate, method of administration, assessment of chocolate intake, and compared with low-flavanol chocolate or placebo or not, will be included.

#### Outcome

The main outcome will be risk of preeclampsia. Preeclampsia will be defined according to the National Heart, Lung, and Blood Institute (NHLBI) guidelines. Risk of preeclampsia would be the ideal outcome measure, but it is likely that there may be observational or interventional studies in pregnant women with pregnancy-induced hypertension, which would be a stage very close to preeclampsia. Therefore, in this review, pregnancy-induced hypertension will be included as a stand-in outcome if the studies measure this outcome. We will include studies only if they measure these outcomes.

#### Search method for identification of studies

Electronic searches of literature will be carried out from their first available date without restriction. PubMed (journal articles from the 1950s onwards), Embase (records from 1974 onwards), and the entire Cochrane Library (2012, issue 12) will be searched. Electronic databases will be searched using a strategy combining selected thesaurus MeSH terms and free text words including chocolate, preeclampsia, pregnant women, and their synonyms [Annex 1 in Additional file 1] for potentially eligible studies. Using the appropriate controlled vocabulary for the search strategies, we will express them in different words as applicable to the databases of PubMed [Annex 2 in Additional file 1], Embase [Annex 3 in Additional file 1], and Cochrane Library [Annex 4 in Additional file 1]. No language restrictions will be used.

## Methods of the review

### Screening

After searching all source databases, all the citations will be exported into EndNote (Carlsbad, CA, USA) software. The accumulated citations will be screened electronically using Microsoft Excel (Redmond, WA, USA). The relevant articles will be obtained following removal of duplicates of the records identified through database searching. Two reviewers (JAM and CB) will independently assess titles and abstracts of pertinent articles found according to the inclusion and exclusion criteria. Previously, we developed a study selection sheet containing titles, abstracts, allocated inclusion status (‘e’, excluded; ‘i’, included; and ‘?’, unclear), and reasons for exclusion. Judgment concerning inclusion will be made individually and results compared. Divergences will be discussed by two authors (JAM and CB) to resolve the conflict; if no agreement is reached, a third reviewer (SD) will be consulted to make a final decision. Finally, full texts of all articles preliminarily identified as unclear for inclusion based on title and abstract will be obtained for a second screen.

### Data extraction

The final set of studies for inclusion will be obtained after review of full-text articles considered eligible by two reviewers (JAM and CB). Data will be extracted through a standardized data compilation form [Annex 5 in Additional file 1] inspired from the Cochrane Collaboration’s tool and then cross-checked. Author names, sample sizes, and results of studies will be compared to avoid duplicates. This form was pilot-tested on three studies to refine it consequently. The data extraction form provides information on qualitative aspects of studies.

Data will be extracted on: 1) publication source (such as date of publication, design, geographical origin, contact details, and location of research group); 2) eligibility verification (such as study design, population, intervention, outcome, and reasons for exclusion); 3) methods of recruitment (such as duration of enrollment, inclusion and exclusion criteria, diagnostic criteria of preeclampsia, study size, and setting); 4) participant characteristics (such as number included in the analysis, age range, mean age, percentage of subjects per age interval, ethnicity, level of education, socioeconomic status, parity, percentage of women by body mass index (BMI) category, smoking status, and percentage of women affected by gestational diabetes); 5) characteristics of intervention evaluated (such as type, timing of exposure, method and technique of measurement, assessment of chocolate consumption, total women by number of servings of chocolate consumed per week by trimester, and assessment of consumption or median theobromine concentration by trimester and theobromine cord concentration); and 6) information of the reported outcome (such as outcome assessed, number of cases with preeclampsia, total cases by quartiles of theobromine concentrations and categories of chocolate consumption by trimester, measures of disease association (crude and adjusted) by quartiles of theobromine concentration, and categories of chocolate consumption by trimester).

### Quality assessment

Two independent reviewers (JAM and CB) will evaluate the risk of bias for eligible studies to ascertain their validity. For this purpose, a standardized form [Annex 6 in Additional file 1] was constructed inspired from the Cochrane Collaboration’s tool for assessing risk of bias, with reference to meta-analysis of observational studies in epidemiology (MOOSE) [16], quality assessment tool for systematic reviews of observational studies (QUATSO) [17], and strengthening the reporting of observational studies in epidemiology (STROBE) [18] quality assessment tools.

The possibility of publication bias will be assessed by evaluating a funnel plot of the studies log(RR) or log(OR) against standard error for asymmetry, which can result from the non-publication of small trials with negative results. Since graphical evaluation can be subjective, an adjusted rank correlation test and a regression asymmetry test will also be conducted as formal statistical tests of publication bias. We acknowledge that other factors, such as differences in study quality or true study heterogeneity, could produce asymmetry in funnel plots.

### Summary of study results

The relative risk (RR) of preeclampsia will be the primary measure of treatment effect. If not reported in included studies, the odds ratio (OR) will be considered instead. Crude and adjusted RR and OR will be treated separately; confounding factors will be noted for adjusted measures of association. In the case of data permitting meta-analysis, the authors will be contacted to obtain crude data in order to calculate RR. If databases are not available, the RR or OR will be taken directly from the publications.

### Planned methods of analysis

Chocolate intake will be determined from food frequency questionnaires, and categories of consumption will be considered in the analysis. Serum theobromine, a biomarker for cocoa, will also be considered in the analysis by interval of concentration. The specific categories will be determined according to included studies.

Analysis of all studies meeting the inclusion criteria will be carried out using Review Manager (RevMan) software (version 5.2; The Nordic Cochrane Centre, Copenhagen). For each study, *n* and the proportion of pregnant women with preeclampsia according to the level of chocolate consumption or control group will be entered into the software. For randomized trials, a meta-analysis will be performed if possible by category of intervention (chocolate consumption) and the random effects model will be used. For non-randomized studies, a meta-analysis will be carried out by study design and potential confounding factors. The potential confounding factors to be explored will be age, ethnicity, level of education, parity, BMI, smoking, and gestational diabetes using directed acyclic graphs (DAGs) to determine required adjustments, and subgroup analysis will be used to explore heterogeneity.

### Exploring heterogeneity

We hypothesize that the effect size may differ according to the methodological quality of the studies, and we will explore the variability of study results (heterogeneity) before conducting the analysis. Heterogeneity will be explored by subgroup analysis according to confounding factors found. If applicable, the robustness of the results will be tested by different outcomes (if available) and type of chocolate.

## Discussion

This systematic review will contribute to establish the current state of knowledge concerning the possible association between chocolate consumption and prevention of preeclampsia, in light of the supporting evidence for the pathophysiologically-related cardiovascular diseases. The results of the systematic review will show the study, population, and intervention characteristics related to preeclampsia.

Furthermore, the systematic review will justify if additional experimental trials are necessary to better evaluate the benefits of chocolate consumption on the risk of preeclampsia. If sufficient data can be extracted, we will consider how the findings can be used to guide future studies in this field and to support the hypothesis regarding the preventive effect of chocolate consumption on the risk of preeclampsia. Finally, we will identify sources of heterogeneity across the selected studies.

## Abbreviations

BMI: Body mass index; BP: Blood pressure; CBA: Controlled before and after; DAG: Directed acyclic graph; ITS: Interrupted time series; MOOSE: Meta-analysis of observational studies in epidemiology; NHLBI: National Heart, Lung, and Blood Institute; NO: Nitric oxide; OR: Odds ratio; PICOS: Participants, interventions, comparators, outcomes, and study design; PRISMA: Preferred Reporting Items for Systematic Reviews and Meta-Analyses; quasi-RCT: Quasi-experimental randomized controlled trial; QUATSO: Quality assessment tool for systematic reviews of observational studies; RR: Relative risk; RTC: Randomized controlled trial; STROBE: Strengthening the reporting of observational studies in epidemiology.

## Competing interests

The authors have no conflicts of interest to report.

## Authors’ contributions

JAM, KP, ST, SL, EB, SD, and CLB developed the objectives of the review and established its relevance in relation to the current literature. JAM and CB conducted scoping searches and created the data extraction quality and assessment forms, which were approved by the remaining the authors. JAM and CB piloted the inclusion/exclusion and will be the first and the second reviewer, respectively. SD will be the third reviewer. JAM wrote the first draft of the manuscript under SD’s supervision and with significant input from all other authors. All authors read and approved the final manuscript.

## Authors’ information

JAM is a nutritionist and PhD candidate in nutrition at Université Laval (QC, Canada). CB is a Senior Resident in Dermatology at Université Laval. KP is MD, PhD at University Teaching Hospital, Pointe-A-Pitre, Guadeloupe. ST is Biostatistician at the Clinical Research Platform CHU de Québec and Research Axis of Public Health and Practice-Changing Research (QC, Canada). SL is Professor of Food Science and Nutrition at Université Laval and Researcher at the Institute of Nutrition and Functional Foods (INAF; QC, Canada). CLB is PhD Research Assistant at Université Laval with special skills in systematic reviews. EB is Professor of Obstetrics and Gynecology at Université Laval and Clinician Scientist at the CHU de Québec Research Center with expertise in systematic reviews. SD is Professor of Obstetrics and Gynecology at Université Laval and Clinician Scientist at the CHU de Québec with expertise in systematic reviews.

## Supplementary Material

Additional file 1 **Annex 1.** Search terms for search strategies. **Annex 2.** Search strategy in PubMed. **Annex 3.** Search strategy in Embase. **Annex 4.** Search strategy in the Cochrane Library. **Annex 5.** Data extraction form. **Annex 6.** Assessment of quality. Click here for file
