# Supplementary material for: Consumption of chocolate in pregnant women and risk of preeclampsia: a systematic review
Source: Syst Rev. 2013 Dec 20;2:114. doi: 10.1186/2046-4053-2-114 (PMC3878223; doi:10.1186/2046-4053-2-114)
Supplement: Additional file 1 — Annex 1. Search terms for search strategies. Annex 2. Search strategy in PubMed. Annex 3. Search strategy in Embase. Annex 4. Search strategy in the Cochrane Library. Annex 5. Data extraction form. Annex 6. Assessment of quality. [file 2046-4053-2-114-S1.pdf]

Annexes

Annex 1. Search terms for search strategies

Key concepts identified

	P	I C	O	S
	pregnant women	chocolate / polyphenolic compounds	preeclampsia	studies design
	pregnan*	cocoa	dysfunction endothelium	randomized and non randomized trials
	women pregnancy	theobromine	endothelial function	all observational studies
**OR**		flavonoids	cardiovascular disease	
		flavanol-rich	hypertension	
		catechin	nitric oxide	
		epicatechin	blood pressure	
		**----- AND ---->**		

Strategy combining

Concepts identified	Other words and spelling variations	
Pregnant women	Synonyms MeSH	
	• Pregnant woman	• Woman, pregnant
	• Women, pregnant	• Pregnancy
	• Pregnance	• Pregnancies
	• Maternity	• Maternal
	• Maternally	• Maternalism
	• Prenatal	• Delivery
	• Perinatal	• Perinatally
	• Puerperium	• Postnatal
	• Accouchement	• Obstetrical delivery

Chocolate / polyphenolic compounds	**Table 1**

• Cacaos	• Theobroma cacao
• Theobroma cacaos	• Cacao, theobroma
• Cacaos, Theobroma	• Cocoa
• Cocoas	• Chocolate
• Chocolates	•

Table 2

• Theobromine	• 3,7-Dimethylxanthine
• Flavanol-rich	• 2-Phenyl-Chromenes
• Flavonoids	• 2-Phenyl-Benzopyrans
• Catechin	• Epicatechin

Catechin and epicatechin

Table 3

• (+)-Cyanidanol	• Cianidanol
• Catechinic Acid	• Acid, Catechinic
• Catechuic Acid	• Acid, Catechuic
• (+)-Catechin	• 3,3',4',5,7-Flavanpentol
• Catergen	• Zyma
• Epicatechin	• 2H-1-Benzopyran-3,5,7-triol, 2-(3,4-dihydroxyphenyl)-3,4-dihydro-, (2R-cis)-
• KB-53	• KB 53
• KB53	• Z 7300
• (+)-Cyanidanol-3	• Cyanidanol-3
• Cyanidanol 3	•

Preeclampsia	**Table 4**

• Pre Eclampsia	• EPH Complex
• EPH Gestosis	• EPH Toxemias
• EPH Toxemia	• Toxemia, EPH
• Toxemias, EPH	• Toxemias, Pregnancy
• Hypertension-Edema-Proteinuria Gestosis	• Gestosis, Hypertension-Edema-Proteinuria
• Hypertension Edema Proteinuria Gestosis	• Preeclampsia
• Pregnancy Toxemias	• Pregnancy Toxemia
• Toxemia, Pregnancy	• Proteinuria-Edema-Hypertension Gestosis
• Gestosis, Proteinuria-Edema-Hypertension	• Proteinuria Edema Hypertension Gestosis
• Edema-Proteinuria-Hypertension Gestosis	• Edema Proteinuria Hypertension Gestosis
• Gestosis, Edema-Proteinuria-Hypertension	• Gestosis, EPH

Complications of pregnancy

Table 5

• Hypertension, Pregnancy Induced	• Pregnancy-Induced Hypertension
• Pregnancy Induced Hypertension	• Hypertensions, Pregnancy Induced
• Induced Hypertension, Pregnancy	• Induced Hypertensions, Pregnancy
• Gestational Hypertension	• Hypertension, Gestational
• Transient Hypertension, Pregnancy	• Hypertension, Pregnancy Transient
• Pregnancy Transient Hypertension	•

Cardiovascular disease

Table 6

• Complications, Cardiovascular Pregnancy	• Pregnancy, Cardiovascular Complications
• Pregnancies, Cardiovascular Complications	• Cardiovascular Pregnancy Complications
• Cardiovascular Pregnancy Complication	• Complication, Cardiovascular Pregnancy
• Pregnancy Complication, Cardiovascular	•

Endothelial function

Table 7

• Endothelium	• Endotheliums

Blood pressure

Table 8

• Pressure, Blood	• Systolic Pressure
• Pressure, Systolic	• Pressures, Systolic
• Diastolic Pressure	• Pressure, Diastolic
• Pulse Pressure	• Pressure, Pulse

Hypertension

Table 9

• Blood Pressure, High	• Blood Pressures, High
• High Blood Pressure	• High Blood Pressures

Nitric Oxide

Table 10

• Oxide, Nitric	• Mononitrogen Monoxide
• Monoxide, Mononitrogen	• Nitrogen Monoxide
• Monoxide, Nitrogen	• Endogenous Nitrate Vasodilator
• Nitrate Vasodilator, Endogenous	• Vasodilator, Endogenous Nitrate
• Nitric Oxide, Endothelium-Derived	• Endothelium-Derived Nitric Oxide
• Nitric Oxide, Endothelium Derived	•

Annex 2. Search strategy in PUBMED

Pregnant women

(Pregnan*[Mesh] OR Pregnant*[tiab] OR (“Pregnant woman” OR Woman, pregnant OR “Women, pregnant” OR Pregnant* OR Pregnancy OR Pregnancies OR Maternity OR Maternal OR Maternally OR “Maternalism” OR Prenatal OR Delivery OR Perinatal OR Perinatally OR puerperium OR Postnatal OR accouchement OR Obstetrical delivery) OR ("Hospitals, Maternity"[Mesh] or Maternity[tiab] or Maternity))

AND

Chocolate / polyphenolic compounds

(("Flavonoids"[Mesh] OR Flavanol-rich[tiab] OR Flavonoids[tiab] OR

"Cacao"[Mesh] OR "Cacao"[TIAB] OR "Theobromine"[Mesh] OR "Theobromine"[TIAB] OR Chocolate[Mesh] OR "Chocolate"[TIAB] OR

"Catechin"[Mesh] OR "Catechin"[TIAB] OR "epicatechin-(4beta-8)-epicatechin-(4beta-8,2beta-O-7)-epicatechin-(4beta-8)-epicatechin" [Supplementary Concept] OR "epicatechin gallate" [Supplementary Concept] OR "epicatechin-(4beta-8,2beta-O-7)-epicatechin-(4beta-8)-epicatechin" [Supplementary Concept] OR "ixoratannin A-2" [Supplementary Concept] OR "epigallocatechin-(4-8,2-O-7)epicatechin" [Supplementary Concept] OR "davallin" [Supplementary Concept] OR "4-(S-cysteinyl)epicatechin 3-O-gallate" [Supplementary Concept] OR "3'-O-cinnamoylprocyanidin A-2" [Supplementary Concept] OR "procyanidin" [Supplementary Concept] OR "epicatechin 3-(3-O-methylgallate)" [Supplementary Concept] OR "epicatechin 5-gallate" [Supplementary Concept] OR "3-O-(3,4,5-trimethoxybenzoyl)epicatechin" [Supplementary Concept] OR "epicatechin-glucuronide" [Supplementary Concept] OR "(4beta-8)-7-O-beta-D-xylopyranosyl-epicatechin" [Supplementary Concept] OR "epicatechin dimer B2" [Supplementary Concept] OR "4-(S-cysteinyl)catechin" [Supplementary Concept] OR "epicatechin-(3'-O-7'')-epiafzelechin" [Supplementary Concept] OR "epicatechin-3'-O-glucuronide" [Supplementary Concept] OR "epicatechin sulfate" [Supplementary Concept] OR "4-(2-aminoethylthio)epicatechin 3-O-gallate" [Supplementary Concept] OR "4-(2-aminoethylthio)epicatechin" [Supplementary Concept] OR "galactopyranosylepicatechin (2-7,4-8)epicatechin" [Supplementary Concept]

OR Cacaos OR Theobroma cacao OR Theobroma cacaos OR cacao, Theobroma OR cacaos, Theobroma OR Cocoa OR Cocoas OR Chocolate OR Chocolates) or (Polypheno* [Mesh] or Polypheno*[tiab] or Polypheno*))

AND

Preeclampsia

(("Pre-Eclampsia"[Mesh]) OR ("Pre-Eclampsia"[TIAB]) OR (Pre-Eclampsia) OR ("Hypertension, Pregnancy-Induced"[Mesh]) OR

("Hypertension"[Mesh]) OR ("Vascular Diseases"[Mesh]) OR ("Cardiovascular Diseases"[Mesh]) OR ("Pregnancy Complications, Cardiovascular"[Mesh]) OR ("Hypertension"[TIAB]) OR ("Vascular Diseases"[TIAB]) OR ("Cardiovascular Diseases"[TIAB]) OR

(Pre Eclampsia OR EPH Complex OR EPH Gestosis OR EPH Toxemias OR EPH Toxemia OR Toxemia, EPH OR Toxemias, EPH OR Toxemias, Pregnancy OR Hypertension-Edema-Proteinuria Gestosis OR Gestosis, Hypertension-Edema-Proteinuria OR Hypertension Edema Proteinuria Gestosis OR Pregnancy Toxemias OR Pregnancy Toxemia OR Toxemia, Pregnancy OR Proteinuria-Edema-Hypertension Gestosis OR Gestosis, Proteinuria-Edema-Hypertension OR Proteinuria Edema Hypertension Gestosis OR Edema-Proteinuria-Hypertension Gestosis OR Edema Proteinuria Hypertension Gestosis OR Gestosis, Edema-Proteinuria-Hypertension OR Gestosis, EPH) OR

(Hypertension, Pregnancy Induced OR Pregnancy-Induced Hypertension OR Pregnancy Induced Hypertension OR Hypertension, Pregnancy Induced OR Induced Hypertension, Pregnancy OR Induced Hypertension, Pregnancy OR Gestational Hypertension OR Hypertension, Gestational OR Transient Hypertension, Pregnancy OR Hypertension, Pregnancy Transient OR Pregnancy Transient Hypertension) OR ("Cardiovascular Diseases"[Mesh] or "Cardiovascular Diseases"[tiab] or "Cardiovascular Diseases") OR (Hypertension[Mesh] or Hypertension[tiab] or Hypertension) OR

("Endothelium, Vascular"[Mesh]) OR (endothelium[tiab]) OR (endothelium OR endotheliums) OR "Hypertension"[Mesh] OR "Hypertension"[TIAB] OR (pressure, blood OR systolic pressure OR pressure, systolic OR pressures, systolic OR diastolic pressure OR pressure, diastolic OR pulse pressure OR pressure, pulse) OR (blood pressure, high OR blood pressures, high OR high blood pressure OR high blood pressures) OR

("Nitric Oxide"[Mesh]) OR ("Nitric Oxide"[TIAB]) OR ("Nitric Oxide Synthase"[Mesh]) OR ("Nitric Oxide Synthase"[tiab]) OR

(Oxide, Nitric OR Mononitrogen Monoxide OR Monoxide, Mononitrogen OR Nitrogen Monoxide OR Monoxide, Nitrogen OR Endogenous Nitrate Vasodilator OR Nitrate Vasodilator, Endogenous OR Vasodilator, Endogenous Nitrate OR Nitric Oxide, Endothelium-Derived OR Endothelium-Derived Nitric Oxide OR Nitric Oxide, Endothelium Derived))

Annex 3. Search strategy in Embase

No.	Query
#32	**#11** AND **#20** AND **#31**
#31	**#21** OR **#22** OR **#23** OR **#24** OR **#25** OR **#26** OR **#27** OR **#28** OR **#29** OR **#30**
#30	**'cardiovascular disease'**/exp OR **'cardiovascular diseases'**
#29	**'nitric oxide'**/exp OR **'nitric oxide'**
#28	**high** AND (**'blood'**/exp OR **blood**) AND (**'pressure'**/exp OR **pressure**)
#27	**'hypertension'**/exp OR **hypertension**
#26	**'blood'**/exp OR **blood** AND (**'pressure'**/exp OR **pressure**)
#25	**'endothelium'**/exp OR **endothelium**
#24	**cardiovascular** AND (**'complication'**/exp OR **complication**) AND (**'pregnancy'**/exp OR **pregnancy**)
#23	**gestational** AND (**'hypertension'**/exp OR **hypertension**)
#22	**'hypertension pregnancy induced'**/exp OR **'hypertension pregnancy induced'**
#21	**'preeclampsia'**/exp OR **preeclampsia**
#20	**#12** OR **#14** OR **#15** OR **#16** OR **#17** OR **#18** OR **#19**
#19	**maternally**
#18	**'delivery'**/exp OR **delivery**
#17	**accouchement**
#16	**'obstetrics'**/exp OR **obstetrics**
#15	**'puerperium'**/exp OR **puerperium**
#14	**maternity**
#12	**pregnan***
#11	**#3** OR **#4** OR **#6** OR **#8** OR **#9** OR **#10**
#10	**'epicatechin'**/exp OR **epicatechin**
#9	**'catechin'**/exp OR **catechin**
#8	**flavano*** OR **polyphenol**
#6	**'theobromine'**/exp OR **theobromine**
#4	**'chocolate'**/exp OR **chocolate**
#3	**'cacao'**/exp OR **cacao**

Annex 4. Search strategy in COCHRANE

ID	Search
**#1**	MeSH descriptor: [Cacao] explode all trees
**#2**	MeSH descriptor: [Theobromine] explode all trees
**#3**	MeSH descriptor: [Flavanones] explode all trees
**#4**	MeSH descriptor: [Catechin] explode all trees
**#5**	MeSH descriptor: [Polyphenols] explode all trees
**#6**	#1 or #2 or #3 or #4 or #5
**#7**	MeSH descriptor: [Pregnant Women] explode all trees
**#8**	MeSH descriptor: [Pregnancy] explode all trees
**#9**	MeSH descriptor: [Hospitals, Maternity] explode all trees
**#10**	MeSH descriptor: [Puerperal Disorders] explode all trees
**#11**	MeSH descriptor: [Delivery, Obstetric] explode all trees
**#12**	#7 or #8 or #9 or #10 or #11
**#13**	MeSH descriptor: [Pre-Eclampsia] explode all trees
**#14**	MeSH descriptor: [Hypertension] explode all trees
**#15**	MeSH descriptor: [Hypertension, Pregnancy-Induced] explode all trees
**#16**	MeSH descriptor: [Cardiovascular Diseases] explode all trees
**#17**	MeSH descriptor: [Blood Pressure] explode all trees
**#18**	MeSH descriptor: [Endothelium] explode all trees
**#19**	MeSH descriptor: [Hypertension] explode all trees
**#20**	MeSH descriptor: [Nitric Oxide] explode all trees
**#21**	#13 or #14 or #15 or #16 or #17 or #18 or #19 or #20
**#22**	#6 and #12 and #21
**#23**	#6 and #12
**#24**	#6 and #21
**#25**	#12 and #21

Annex 5 Data extraction form

1. Publication source

No.	Variable	Variable description	Options	Class
**1.1**	IDscreening	ID number of first screening	(number)	Continuous
**1.2**	StudyID	ID number data extraction	(number)	Continuous
**1.3**	Title	Study title of first screening	(full title)	Nominal
**1.4**	Authors	Names of authors	(last name first author, initials first name)	Nominal
**1.5**	Year	Year of study publishing	(year in numbers)	Continuous
**1.6**	ReviewID	Number of review author identification	(number)	Continuous
**1.7**	Contact	Contact details	(name and email contact author; non-reported)	Nominal
**1.8**	Journal	Publication journal	(full name)	Nominal
**1.9**	City	Publication city	(full name)	Nominal
**1.10**	Country	Publication country	(full name)	Nominal

2. Eligibility verification

No.	Variable	Variable description	Options	Class
**2.1**	StudyID	ID number data extraction	(number)	Continuous
**2.2**	Design	Study design	(design)	Nominal
**2.3**	Population	Pregnant women	(Y:Yes, N:Not)	Dichotomous
**2.4**	Intervention	Chocolate consumption	(Y:Yes, N:Not)	Dichotomous
**2.5**	Outcome	Preeclampsia	(Y:Yes, N:Not)	Dichotomous
**2.6**	Eligibility	Verification of eligibility	(Y:Yes, N:Not)	Dichotomous
**2.7**	Exclusion	Reasons for exclusion	(population, intervention or outcome)	Nominal

3. Methods

No.	Variable	Variable description	Options	Class
**3.1**	StudyID	ID number data extraction	(number)	Continuous
**3.2**	Design	Study design	(CC: case control; C: cohort; RCT: randomized	Nominal

			control trial; T: transversal; non-reported)	
**3.3**	Details of duration			
**3.3.1**	Beginning	Date of beginning enrollment	(Date: mm-yyyy)	Date
**3.3.2**	End	Date of ending enrollment	(Date: mm-yyyy)	Date
**3.3.3**	Duration	Duration of enrollment in months	(number)	Continuous
**3.4**	Inclusion criteria			
**3.4.1**	Singleton	Singleton pregnancy	(Y:Yes, N:No)	Dichotomous
**3.4.2**	Liveborn	Birth of a live infant	(Y:Yes, N:No)	Dichotomous
**3.5**	Exclusion criteria			
**3.5.1**	CHT	Presence of chronic hypertension	(Y:Yes, N:No)	Dichotomous
**3.5.2**	CRD	Presence of chronic renal disease	(Y:Yes, N:No)	Dichotomous
**3.5.3**	Diabetes	Presence of diabetes	(Y:Yes, N:No)	Dichotomous
**3.5.4**	Twin	Presence of a twin gestation	(Y:Yes, N:No)	Dichotomous
**3.6**	Diagnostic criteria of preeclampsia			
**3.6.1**	Hypertension	Diagnostic criteria regarding hypertension of preeclampsia	(name)	Nominal
**3.6.2**	Proteinuria	Diagnostic criteria regarding proteinuria of preeclampsia	(name)	Nominal
**3.6.3**	Other	Other diagnostic criteria considered	(name)	Nominal
**3.7**	Study size			
**3.7.1**	Screened	Number of subjects screened for eligibility	(number)	Continuous
**3.7.2**	PotEligible	Number of potentially eligible subjects	(number)	Continuous
**3.7.3**	Included	Number of subjects included in the study	(number)	Continuous
**3.7.4**	AnalysedC	Number of subjects analysed with regard to chocolate consumption	(number)	Continuous
**3.7.5**	AnalysedT	Number of subjects analysed with regard to serum theobromine concentration	(number)	Continuous
**3.8**	Setting	Setting of care	(characteristics)	Nominal

4. Participants

No.	Variable	Variable description	Options	Class
**4.1**	StudyID	ID number data extraction	(number)	Continuous
**4.2**	City	Participant city	(name)	Nominal
**4.3**	Country	Participant country	(name)	Nominal
**4.4**	Percentage of subjects per age interval			
**4.4.1**	≤24	Percentage of subjects of 24 years of age or under	(number)	Categorical

4.4.2	25-29	Percentage of subjects of 25-29 years of age	(number)	Categorical
4.4.3	30-34	Percentage of subjects of 30-34 years of age	(number)	Categorical
4.4.4	>35	Percentage of subjects over 35 years of age	(number)	Categorical
**4.5**	Percentage of women by ethnicity			
**4.5.1**	White	Percentage of women of white ethnicity	(number)	Categorical
**4.5.2**	Non-white	Percentage of women of non-white ethnicity	(number)	Categorical
**4.6**	Percentage of women by level of education			
**4.6.1**	≤12	Percentage of women with 12 years or less of education, corresponding to High School education or less	(number)	Categorical
**4.6.2**	>12	Percentage of women with more than 12 years of education, corresponding to any education higher than High School	(number)	Categorical
**4.7**	Percentage of women by parity			
**4.7.1**	Nulliparous	Percentage of nulliparous women	(number)	Categorical
**4.7.2**	Parous	Percentage of parous women	(number)	Categorical
**4.8**	Percentage of women by body mass index category			
**4.8.1**	Underweight	Percentage of women of underweight BMI	(number)	Categorical
**4.8.2**	Normal	Percentage of women of normal BMI	(number)	Categorical
**4.8.3**	Overweight	Percentage of women of overweight BMI	(number)	Categorical
**4.8.4**	Obese	Percentage of women of obese BMI	(number)	Categorical
**4.9**	Percentage of women by smoking status			
**4.9.1**	Non-smoker	Percentage of women reporting not to smoke	(number)	Categorical
**4.9.2**	Smoker	Percentage of women reporting to be smokers	(number)	Categorical
**4.10**	Percentage of women affected by gestational diabetes			
**4.10.1**	Diabetic	Percentage of women affected by gestational diabetes	(number)	Categorical
**4.10.2**	Non-diabetic	Percentage of women not affected by gestational diabetes	(number)	Categorical

5. Interventions

No.	Variable	Variable description	Options	Class
**5.1**	StudyID	ID number data extraction	(number)	Continuous

**5.2**	Exposure	Exposure assessment	(name)	Nominal
**5.3**	TimingF	Timing of exposure in 1st trimester evaluated in days	(number)	Continuous
**5.4**	TimingT	Timing of exposure in 3rd trimester evaluated in days	(number)	Continuous
**5.5**	Technique	Technique used for quantification of theobromine concentration in serum	(name)	Nominal
**5.6**	Detection	Limit of detection of theobromine (ng/dl)	(number)	Continuous
**5.7**	Quantification	Limit of quantification of theobromine (ng/dl)	(number)	Continuous
**5.8**	Variation	Intra-assay coefficient of variation (ng/dl)	(number)	Continuous
**5.9**	Theobromine concentration in 1st trimester			
**5.9.1**	MedianF	Median theobromine concentration in 1st trimester	(number)	Continuous
**5.9.2**	Quartile1F	1st quartile of theobromine concentration in 1st trimester	(interval)	Interval
**5.9.3**	Quartile2F	2nd quartile of theobromine concentration in 1st trimester	(interval)	Interval
**5.9.4**	Quartile3F	3rd quartile of theobromine concentration in 1st trimester	(interval)	Interval
**5.9.5**	Quartile4F	4th quartile of theobromine concentration in 1st trimester	(interval)	Interval
**5.10**	Theobromine concentration in 3rd trimester			
**5.10.1**	MedianT	Median theobromine concentration in 3rd trimester	(number)	Continuous
**5.10.2**	Quartile1T	1st quartile of theobromine concentration in 3rd trimester	(interval)	Interval
**5.10.3**	Quartile2T	2nd quartile of theobromine concentration in 3rd trimester	(interval)	Interval
**5.10.4**	Quartile3T	3rd quartile of theobromine concentration in 3rd trimester	(interval)	Interval
**5.10.5**	Quartile4T	4th quartile of theobromine concentration in 3rd trimester	(interval)	Interval
**5.11**	Total women by theobromine quartile in 1st trimester			
**5.11.1**	TQuartile1F	Total women in 1st quartile of theobromine concentration in 1st trimester	(number)	Categorical
**5.11.2**	TQuartile2F	Total women in 2nd quartile of theobromine concentration in 1st trimester	(number)	Categorical
**5.11.3**	TQuartile3F	Total women in 3rd quartile of theobromine concentration in 1st trimester	(number)	Categorical
**5.11.4**	TQuartile4F	Total women in 4th quartile of theobromine concentration in 1st trimester	(number)	Categorical
**5.12**	Total women by theobromine quartile in 3rd trimester			
**5.12.1**	TQuartile1T	Total women in 1st quartile of theobromine concentration in 3rd	(number)	Categorical

		trimester		
**5.12.2**	TQuartile2T	Total women in 2nd quartile of theobromine concentration in 3rd trimester	(number)	Categorical
**5.12.3**	TQuartile3T	Total women in 3rd quartile of theobromine concentration in 3rd trimester	(number)	Categorical
**5.12.4**	TQuartile4T	Total women in 4th quartile of theobromine concentration in 3rd trimester	(number)	Categorical
**5.13**	Chocolate	Assessment of chocolate consumption	(Y:Yes, N:No)	Dichotomous
**5.14**	Total women by number of servings of chocolate drinks and foods consumed per week in 1st trimester			
**5.14.1**	T<1F	Less than 1 serving of chocolate drinks and foods consumed per week in 1st trimester	(number)	Categorical
**5.14.2**	ToccF	Occasional consumption of chocolate drinks and foods in 1st trimester (1 to 3-4 servings per week)	(number)	Categorical
**5.14.3**	TregF	Regular consumption of chocolate drinks and foods in 1st trimester (4-5 or more servings per week)	(number)	Categorical
**5.15**	Total women by number of servings of chocolate drinks and foods consumed per week in 3rd trimester			
**5.15.1**	T<1T	Less than 1 serving of chocolate drinks and foods consumed per week in 3rd trimester	(number)	Categorical
**5.15.2**	ToccT	Occasional consumption of chocolate drinks and foods in 3rd trimester (1 to 3-4 servings per week)	(number)	Categorical
**5.15.3**	TregT	Regular consumption of chocolate drinks and foods in 3rd trimester (4-5 or more servings per week)	(number)	Categorical
**5.16**	Source	Source of chocolate	(name)	Nominal
**5.17**	Caffeine	Assessment of consumption or cord concentration	(Y:Yes, N:No)	Dichotomous

6. Outcomes

No.	Variable	Variable description	Options	Class
**6.1**	StudyID	ID number data extraction	(number)	Continuous
**6.2**	Preeclampsia	Outcome assessed	(Y:Yes, N:No)	Dichotomous
**6.3**	Cases	Number of cases with preeclampsia	(number)	Continuous
**6.4**	Total cases by quartiles of theobromine concentrations in 1st trimester			
**6.4.1**	CQuartile1F	Total cases in 1st quartile of theobromine concentration in 1st trimester	(number)	Categorial

**6.4.2**	CQuartile2F	Total cases in 2nd quartile of theobromine concentration in 1st trimester	(number)	Categorial
**6.4.3**	CQuartile3F	Total cases in 3rd quartile of theobromine concentration in 1st trimester	(number)	Categorial
**6.4.4**	CQuartile4F	Total cases in 4th quartile of theobromine concentration in 1st trimester	(number)	Categorial
**6.5**	Total cases by quartiles of theobromine concentrations in 3rd trimester			
**6.5.1**	CQuartile1T	Total cases in 1st quartile of theobromine concentration in 3rd trimester	(number)	Categorial
**6.5.2**	CQuartile2T	Total cases in 2nd quartile of theobromine concentration in 3rd trimester	(number)	Categorial
**6.5.3**	CQuartile3T	Total cases in 3rd quartile of theobromine concentration in 3rd trimester	(number)	Categorial
**6.5.4**	CQuartile4T	Total cases in 4th quartile of theobromine concentration in 3rd trimester	(number)	Categorial
**6.6**	Total cases by categories of chocolate consumption in 1st trimester			
**6.6.1**	C<1F	Total cases for less than 1 serving of chocolate drinks and foods consumed per week in 1st trimester	(number)	Categorial
**6.6.2**	CoccF	Total cases for occasional consumption of chocolate drinks and foods (1 to 3-4 servings per week) in 1st trimester	(number)	Categorial
**6.6.3**	CregF	Total cases for regular consumption of chocolate drinks and foods (4-5 or more servings per week) in 1st trimester	(number)	Categorial
**6.7**	Total cases by categories of chocolate consumption in 3rd trimester			
**6.7.1**	C<1T	Total cases for less than 1 serving of chocolate drinks and foods consumed per week in 3rd trimester	(number)	Categorial
**6.7.2**	CoccT	Total cases for occasional consumption of chocolate drinks and foods (1 to 3-4 servings per week) in 3rd trimester	(number)	Categorial
**6.7.3**	CregT	Total cases for regular consumption of chocolate drinks and foods (4-5 or more servings per week) in 3rd trimester	(number)	Categorial
**6.8**	OR by quartiles of theobromine concentration			
**6.8.1**	ORQuartile1	OR for 1st quartile of theobromine concentration	(number)	Continuous
**6.8.2**	ORQuartile2	OR for 2nd quartile of theobromine concentration	(number)	Continuous
**6.8.3**	ORQuartile3	OR for 3rd quartile of theobromine concentration	(number)	Continuous
**6.8.4**	ORQuartile4	OR for 4th quartile of theobromine concentration	(number)	Continuous

**6.9**	Ajusted OR by quartiles of theobromine concentration			
**6.9.1**	aORQuartile1	Ajusted OR for 1st quartile of theobromine concentration	(number)	Continuous
**6.9.2**	aORQuartile2	Ajusted OR for 2nd quartile of theobromine concentration	(number)	Continuous
**6.9.3**	aORQuartile3	Ajusted OR for 3rd quartile of theobromine concentration	(number)	Continuous
**6.9.4**	aORQuartile4	Ajusted OR for 4th quartile of theobromine concentration	(number)	Continuous
**6.10**	OR by selected values of theobromine concentration in 1st trimester			
**6.10.1**	OR100F	OR for 100 ng/ml in 1st trimester	(number)	Continuous
**6.10.2**	OR200F	OR for 200 ng/ml in 1st trimester	(number)	Continuous
**6.10.3**	OR300F	OR for 300 ng/ml in 1st trimester	(number)	Continuous
**6.10.4**	OR500F	OR for 500 ng/ml in 1st trimester	(number)	Continuous
**6.10.5**	OR700F	OR for 700 ng/ml in 1st trimester	(number)	Continuous
**6.10.6**	OR900F	OR for 900 ng/ml in 1st trimester	(number)	Continuous
**6.10.7**	OR1500F	OR for 1500 ng/ml in 1st trimester	(number)	Continuous
**6.11**	Ajusted OR by selected values of theobromine concentration in 1st trimester			
**6.11.1**	aOR100F	OR for 100 ng/ml in 1st trimester	(number)	Continuous
**6.11.2**	aOR200F	OR for 200 ng/ml in 1st trimester	(number)	Continuous
**6.11.3**	aOR300F	OR for 300 ng/ml in 1st trimester	(number)	Continuous
**6.11.4**	aOR500F	OR for 500 ng/ml in 1st trimester	(number)	Continuous
**6.11.5**	aOR700F	OR for 700 ng/ml in 1st trimester	(number)	Continuous
**6.11.6**	aOR900F	OR for 900 ng/ml in 1st trimester	(number)	Continuous
**6.11.7**	aOR1500F	OR for 1500 ng/ml in 1st trimester	(number)	Continuous
**6.12**	OR by selected values of theobromine concentration in 3rd trimester			
**6.12.1**	OR100T	OR for 100 ng/ml in 3rd trimester	(number)	Continuous
**6.12.2**	OR200T	OR for 200 ng/ml in 3rd trimester	(number)	Continuous
**6.12.3**	OR300T	OR for 300 ng/ml in 3rd trimester	(number)	Continuous
**6.12.4**	OR500T	OR for 500 ng/ml in 3rd trimester	(number)	Continuous
**6.12.5**	OR700T	OR for 700 ng/ml in 3rd trimester	(number)	Continuous
**6.12.6**	OR900T	OR for 900 ng/ml in 3rd trimester	(number)	Continuous
**6.12.7**	OR1500T	OR for 1500 ng/ml in 3rd trimester	(number)	Continuous
**6.13**	Ajusted OR by selected values of theobromine concentration in 3rd trimester			
**6.13.1**	aOR100T	OR for 100 ng/ml in 3rd trimester	(number)	Continuous
**6.13.2**	aOR200T	OR for 200 ng/ml in 3rd trimester	(number)	Continuous
**6.13.3**	aOR300T	OR for 300 ng/ml in 3rd trimester	(number)	Continuous
**6.13.4**	aOR500T	OR for 500 ng/ml in 3rd trimester	(number)	Continuous
**6.13.5**	aOR700T	OR for 700 ng/ml in 3rd trimester	(number)	Continuous
**6.13.6**	aOR900T	OR for 900 ng/ml in 3rd trimester	(number)	Continuous
**6.13.7**	aOR1500T	OR for 1500 ng/ml in 3rd trimester	(number)	Continuous
**6.14**	OR by categories of chocolate consumption in 1st trimester			
**6.14.1**	OR<1F	OR for less than 1 serving of chocolate drinks and foods consumed per week in 1st trimester	(number)	Continuous

**6.14.2**	ORoccF	OR for occasional consumption of chocolate drinks and foods (1 to 3-4 servings per week) in 1st trimester	(number)	Continuous
**6.14.3**	ORregF	OR for regular consumption of chocolate drinks and foods (4-5 or more servings per week) in 1st trimester	(number)	Continuous
**6.15**	Ajusted OR by categories of chocolate consumption in 1st trimester			
**6.15.1**	aOR<1F	Ajusted OR for less than 1 serving of chocolate drinks and foods consumed per week in 1st trimester	(number)	Continuous
**6.15.2**	AORoccF	Ajusted OR for occasional consumption of chocolate drinks and foods (1 to 3-4 servings per week) in 1st trimester	(number)	Continuous
**6.15.3**	AORregF	Ajusted OR for regular consumption of chocolate drinks and foods (4-5 or more servings per week) in 1st trimester	(number)	Continuous
**6.16**	OR by categories of chocolate consumption in 3rd trimester			
**6.16.1**	OR<1T	OR for less than 1 serving of chocolate drinks and foods consumed per week in 3rd trimester	(number)	Continuous
**6.16.2**	ORoccT	OR for occasional consumption of chocolate drinks and foods (1 to 3-4 servings per week) in 3rd trimester	(number)	Continuous
**6.16.3**	ORregT	OR for regular consumption of chocolate drinks and foods (4-5 or more servings per week) in 3rd trimester	(number)	Continuous
**6.17**	Ajusted OR by categories of chocolate consumption in 3rd trimester			
**6.17.1**	aOR<1T	Ajusted OR for less than 1 serving of chocolate drinks and foods consumed per week in 3rd trimester	(number)	Continuous
**6.17.2**	AORoccT	Ajusted OR for occasional consumption of chocolate drinks and foods (1 to 3-4 servings per week) in 3rd trimester	(number)	Continuous
**6.17.3**	AORregT	Ajusted OR for regular consumption of chocolate drinks and foods (4-5 or more servings per week) in 3rd trimester	(number)	Continuous

7. Risk of bias

No.	Variable	Variable description	Options	Class
**7.1**	StudyID	ID number data extraction	(number)	Continuous
**7.2**	Selection quality of participants in the studies			
**7.2.1**	Criteria	Can the criteria for inclusion and exclusion clearly identify the study population?	(Y:Yes, N:Not, U:unclear, NR: non-reported; N/A: non-applicable)	Dichotomous
**7.2.2**	Control	Was an appropriate control group used?	(Y:Yes, N:Not, U:unclear, NR: non-reported; N/A: non-applicable)	Dichotomous

**7.2.3**	Flow	Is the flow of participants explained in detail?	(Y:Yes, N:Not, U:unclear, NR: non-reported; N/A: non-applicable)	Dichotomous
**7.2.4**	Detail	Are the study participants described in detail?	(Y:Yes, N:Not, U:unclear, NR: non-reported; N/A: non-applicable)	Dichotomous
**7.3**	Information quality regarding of exposure evaluation			
**7.3.1**	Consumption	Are objective, suitable, and standard criteria used for measurement of chocolate consumption?	(Y:Yes, N:Not, U:unclear, NR: non-reported; N/A: non-applicable)	Dichotomous
**7.3.2**	Biomarker	Is the biomarker of chocolate consumption used suitable?	(Y:Yes, N:Not, U:unclear, NR: non-reported; N/A: non-applicable)	Dichotomous
**7.3.3**	Categories	Is the exposition categorized consistently throughout the study?	(Y:Yes, N:Not, U:unclear, NR: non-reported; N/A: non-applicable)	Dichotomous
**7.4**	Information quality regarding of outcome evaluation			
**7.4.1**	Preeclampsia	Are objective, suitable, and standard criteria used for measurement of preeclampsia?	(Y:Yes, N:Not, U:unclear, NR: non-reported; N/A: non-applicable)	Dichotomous
**7.4.2**	Blinded	Were investigators collecting information regarding outcome blinded to exposure status?	(Y:Yes, N:Not, U:unclear, NR: non-reported; N/A: non-applicable)	Dichotomous
**7.5**	Choice of design			
**7.5.1**	Appropriate	Is the study design appropriate for the research question?	(Y:Yes, N:Not, U:unclear, NR: non-reported; N/A: non-applicable)	Dichotomous
**7.5.2**	Data	Did the study use data that hadn't been originally collected for another purpose?	(Y:Yes, N:Not, U:unclear, NR: non-reported; N/A: non-applicable)	Dichotomous
**7.6**	Quality of simple size			
**7.6.1**	Size	Is the sample size adequately calculated?	(Y:Yes, N:Not, U:unclear, NR: non-reported; N/A: non-applicable)	Dichotomous
**7.7**	Quality of statistical analyses			
**7.7.1**	Statistical	Are the statistical tests chosen for analysis of the association appropriate?	(Y:Yes, N:Not, U:unclear, NR: non-reported; N/A: non-applicable)	Dichotomous
**7.7.2**	Non-analysed	Are reasons for exclusion of subjects from statistical analysis explained?	(Y:Yes, N:Not, U:unclear, NR: non-reported; N/A: non-applicable)	Dichotomous
**7.7.3**	Confounding	Were confounding variables controlled for in the analysis?	(Y:Yes, N:Not, U:unclear, NR: non-reported; N/A: non-applicable)	Dichotomous
**7.7.4**	Continuous	Do the results clearly report how they had been adjusted for continuous confounders?	(Y:Yes, N:Not, U:unclear, NR: non-reported; N/A: non-applicable)	Dichotomous
**7.7.5**	OR	Are the estimates of risk (odds ratio) given with CIs?	(Y:Yes, N:Not, U:unclear, NR: non-reported; N/A: non-applicable)	Dichotomous

**7.8**	Quality	Overall quality of studies	(L: Low, I:Intermediate, H:High)	Categorical

Annex 6 Assessment of quality

Criteria		Studies		
**Participants**	Can the criteria for inclusion and exclusion clearly identify the study population?			
	Was an appropriate control group used?			
	Is the flow of participants explained in detail?			
	Are the study participants described in detail?			
**Intervention**	Are objective, suitable, and standard criteria used for measurement of chocolate consumption?			
	Is the biomarker of chocolate consumption used suitable?			
	Is the exposition categorized consistently throughout the study?			
**Outcome**	Are objective, suitable, and standard criteria used for measurement of preeclampsia?			
	Were investigators collecting information regarding outcome blinded to exposure status?			
**Design**	Is the study design appropriate for the research question?			
	Did the study use data that hadn't been originally collected for another purpose?			
**Sample size**	Is the sample size adequately calculated?			
**Statistical analyses**	Are the statistical tests chosen for analysis of the association appropriate?			
	Are the reasons for exclusion of subjects from statistical analysis explained?			
	Were confounding variables controlled for in the analysis?			
	Do the results clearly report how they had been adjusted for continuous confounders?			
	Are the estimates of risk (odds ratio) given with CIs?			
**Overall quality**				
